# Reduced ADAMTS13 levels in patients with acute and chronic cerebrovascular disease

**DOI:** 10.1371/journal.pone.0179258

**Published:** 2017-06-07

**Authors:** Frederik Denorme, Peter Kraft, Inge Pareyn, Christiane Drechsler, Hans Deckmyn, Karen Vanhoorelbeke, Christoph Kleinschnitz, Simon F. De Meyer

**Affiliations:** 1 Laboratory for Thrombosis Research, KU Leuven Campus Kulak Kortrijk, Kortrijk, Belgium; 2 Department of Neurology, University Hospital of Würzburg, Würzburg, Germany; 3 Department of Internal Medicine, University Hospital of Würzburg, Würzburg, Germany; 4 Department of Neurology, University Hospital Essen, Essen, Germany; University of Münster, GERMANY

## Abstract

Von Willebrand Factor (VWF) plays a major role in thrombosis and hemostasis and its thrombogenicity is controlled by ADAMTS13. Whereas increasing evidence shows a clear association between VWF levels and acute ischemic stroke, little is known about a correlation with ADAMTS13. Therefore, the aim of this study was to compare plasma levels of ADAMTS13 between 85 healthy volunteers (HV), 104 patients with acute ischemic stroke and 112 patients with a chronic cerebrovascular disease (CCD). In this case-control study, plasma ADAMTS13 antigen levels were measured by ELISA and plasma VWF levels, measured previously, were next used to calculate VWF:ADAMTS13 ratios. ADAMTS13 levels and VWF:ADAMTS13 ratios were subsequently correlated with key demographic and clinical parameters. ADAMTS13 levels were significantly lower in acute ischemic stroke patients (82.6 ± 21.0%) compared with HV (110.6 ± 26.9%). Also, CCD patients (99.6 ± 24.5%) had significantly lower ADAMTS13 levels compared with HV however these were still higher than in acute stroke patients. Furthermore, when assessing the VWF:ADAMTS13 ratios, an even greater difference was revealed between stroke patients (2.7 ± 1.9), HV (1.1 ± 0.5) and CCD patients (1.7 ± 0.7). The VWF:ADAMTS13 ratio was significantly associated with stroke severity and modality. In conclusion, both in acute and chronic cerebrovascular disease patients, ADAMTS13 levels were significantly decreased, with the lowest ADAMTS13 levels found in acute stroke patients. This difference was even more distinct when the ratio of VWF:ADAMTS13 was considered. These results demonstrate the potentially important involvement of the VWF/ADAMTS13 axis in ischemic stroke.

## Introduction

Ischemic stroke is one of the leading causes of death and sustained disability in the Western world. While many risk factors have already been identified, the exact pathogenesis of ischemic stroke remains unclear and treatment options are limited. In recent years, the detrimental role of von Willebrand factor (VWF) in ischaemic stroke has gained increasing attention.[1] VWF is a multimeric plasma glycoprotein that recruits platelets at sites of vascular injury and thereby contributes to thrombosis and hemostasis. By recruiting leukocytes, VWF also promotes inflammation thereby creating a thrombo-inflammatory environment.[2] The activity of VWF is controlled by the metalloproteinase ADAMTS13 (A Disintegrin And Metalloproteinase with Thrombospondin type 1 motif, member 13). ADAMTS13 cleaves ultra-large, highly reactive VWF multimers into smaller, less active ones, preventing spontaneous platelet-thrombus formation and reducing inflammation.[3] Preclinical animal studies have shown that absence of VWF is protective in ischemic stroke whereas absence of ADAMTS13 worsens disease outcome.[4–6] Increasing clinical evidence also demonstrates a clear association between high VWF-levels and acute ischaemic stroke in patients. The possible association between stroke and ADAMTS13 levels is however less studied, with some conflicting results reported.[7] We therefore performed a case-control study to investigate levels of ADAMTS13 in patients during the acute phase of ischemic stroke.

## Material and methods

### Data collection

As cases, patients with acute ischemic stroke (AIS), transitory ischemic attack (TIA)) and chronic cerebrovascular disease (CCD) were enrolled.[8] Participants were recruited at the Stroke Unit (patients diagnosed with TIA or AIS), at the outpatient clinic for CCD, or after a call for healthy volunteers (HV) at the Neurology Department, University Hospital of Würzburg (Germany) between September 2010 and January 2013. Inclusion criteria included blood withdrawal within 24 h after symptom onset in AIS (defined as acute ischemic lesion on brain imaging) or TIA (no acute lesion) patients, presentation with extra- and/or intracranial stenosis of the large cerebral arteries with (n = 67) or without (n = 45) history of AIS or TIA for the CCD group and no history of stroke, myocardial infarction, or peripheral arterial disease for the HV group. Furthermore, HV had to be older than 50 years. Exclusion criteria were hemorrhagic stroke, age < 18 years, and known platelet dysfunction or plasmatic coagulation disorders based on a detailed medical history and collection of routine coagulation parameters. Only non-hemolyzed blood samples were analyzed. Overall, 104 patients with stroke (AIS or TIA), 112 patients with CCD, and 85 HV fulfilled the inclusion criteria and took part in the study. In the patients with AIS or TIA, TOAST (Trial of Org 10172 in Acute Stroke Treatment) criteria were applied in an adapted form: (1) cardio-embolism, (2) large-artery atherosclerosis, (3) small-vessel occlusion, or (4) other determined or undetermined etiology. The National Institute of Health Stroke Scale (NIHSS) and Barthel Index (BI) score and modified Ranking Scale (mRS) were determined at patient admission. The latency between symptom onset and blood withdrawal, platelet inhibitor pre-treatment and modality of acute stroke therapy (thrombolysis vs. no thrombolysis) were registered. Platelet inhibitors included aspirin, dipyridamol, clopidogrel and prasugrel.

### Blood collection

Trisodium citrate anticoagulated blood was collected on day 0, 1, and 3 in patients with stroke, and once in CCD patients and HV between 08.00 and 12.00 hours from an antecubital vein using a 21-gauge butterfly needle.

### Measurement of ADAMTS13 levels

ADAMTS13 levels in plasma samples were measured by an in-house ADAMTS13:Ag ELISA as previously described with minor modifications.[9] In brief, monoclonal anti-ADAMTS-13 antibody 3H9 was coated (5 μg/mL) in a 96-well microtiter plate overnight at 4°C in 0.05 M carbonate/bicarbonate. Bound ADAMTS13 was detected using a mix of biotinylated anti-ADAMTS-13 antibodies 19H4 and 17G2 (1.5 μg/mL each in phosphate buffered saline, 0.3% milk) and HRP-labelled streptavidin (1/10 000 dilution in phosphate buffered saline, 0.3% milk). To compare all plasma samples, a dilution series (in phosphate buffered saline, 0.3% milk) of a normal human plasma (NHP) pool was applied as a standard on each plate. This NHP pool was composed of citrated plasma from 20 healthy donors, recruited at the local laboratory in KU Leuven Campus Kulak Kortrijk, Belgium. ADAMTS13 levels were subsequently expressed as a percentage of NHP ADAMTS13 antigen. VWF:ADAMTS13 ratios were determined for each patient using VWF antigen levels that were measured in a previous study on the same set of plasma samples.[8]

### Statistical analysis

Continuous variables are expressed as mean with standard deviation or as median with interquartile range, as appropriate. The association of ADAMTS13 concentrations and the ratios of VWF:ADAMTS13 were explored with a range of demographic and clinical characteristics: age, sex, neurological scales, disease modality (TIA or AIS), TOAST criteria, duration between symptom onset and blood withdrawal, NIHSS, Barthel Index, treatment modality (intravenous thrombolysis or not), and intake of platelet inhibitors in the days before blood withdrawal. P values for comparisons across groups of clinical and demographic characteristics were derived from analysis of variance (ANOVA), and the chi-square test, as appropriate. ADAMTS13 levels and the ratios of VWF:ADAMTS13 were compared between the different patient groups (AIS/TIA inpatients, CCD outpatients, or HV) by a Kruskal-Wallis test with a Dunn's Multiple comparison test and additionally adjusted for age and sex. Distribution was analyzed using the Kolmogorov–Smirnov test. To identify independent predictors of ADAMTS13 levels and the ratios of VWF:ADAMTS13, a linear regression model was used that included all variables without co-linearity in a multivariate model adjusting for age and sex. Using this model, coefficients and the corresponding 95% confidence intervals (CIs) were estimated. All reported P values are two-sided and a P value < 0.05 was considered statistically significant. Analyses were performed using SPSS Version 21 and SAS software version 9.1 (SAS Institute Inc., Cary, NC).

### Ethics

Informed written consent was obtained from all participants. The study protocol was approved by the ethics committee of the medical faculty of the University of Würzburg, Germany (reference number 65/2010). Study participation had no influence on treatment and patient care.

## Results

### Overall clinical characteristics

A total of 104 acute stroke patients were included in this study. The mean age of these patients was 69 ± 12.5 years and 54.3% were male. The baseline clinical severity measured with NIHSS and Barthel Index was 4.7 ± 6.0 and 75 ± 28, respectively. More than half of the patients had AIS (58%). A detailed descriptive analysis of the characteristics of the patients with acute cerebrovascular event is given in Table 1.

**Table 1:** Baseline characteristics of the acute stroke patients (acute ischemic stroke (AIS) and transient ischemic attack (TIA)). Data are represented as mean ± standard deviation (SD). (NIHSS: National Institutes of Health stroke scale; TOAST: Trial of Org 10172 in Acute Stroke Treatment).

Characteristic	Value
**Age, years (median (range))**	71 (36–96)
**Sex (n (%))**	
Male	57 (54.3)
Female	48 (45.7)
**Modality (n (%))**	
AIS	61 (58.1)
TIA	44 (41.9)
**Modified TOAST criteria (n (%))**	
Cardio embolism	61 (58.1)
Large-artery atherosclerosis	4 (3.8)
Small-vessel occlusion	11 (10.5)
Other or undetermined etiology	29 (27.6)
**Thrombolysis (n (%))**	84 (80)
**Platelet inhibitor before blood withdrawal (n (%))**	66 (62.9)
**NIHSS at admission (mean)**	4.7 ± 6.0
**Barthel Index at admission (mean)**	75 ± 28
**Modified Rankin Scale at admission (mean)**	2.2 ± 1.6

### Lower ADAMTS13 levels and higher VWF:ADAMTS13 ratios in acute and chronic cerebrovascular disease patients

**Fig 1:**
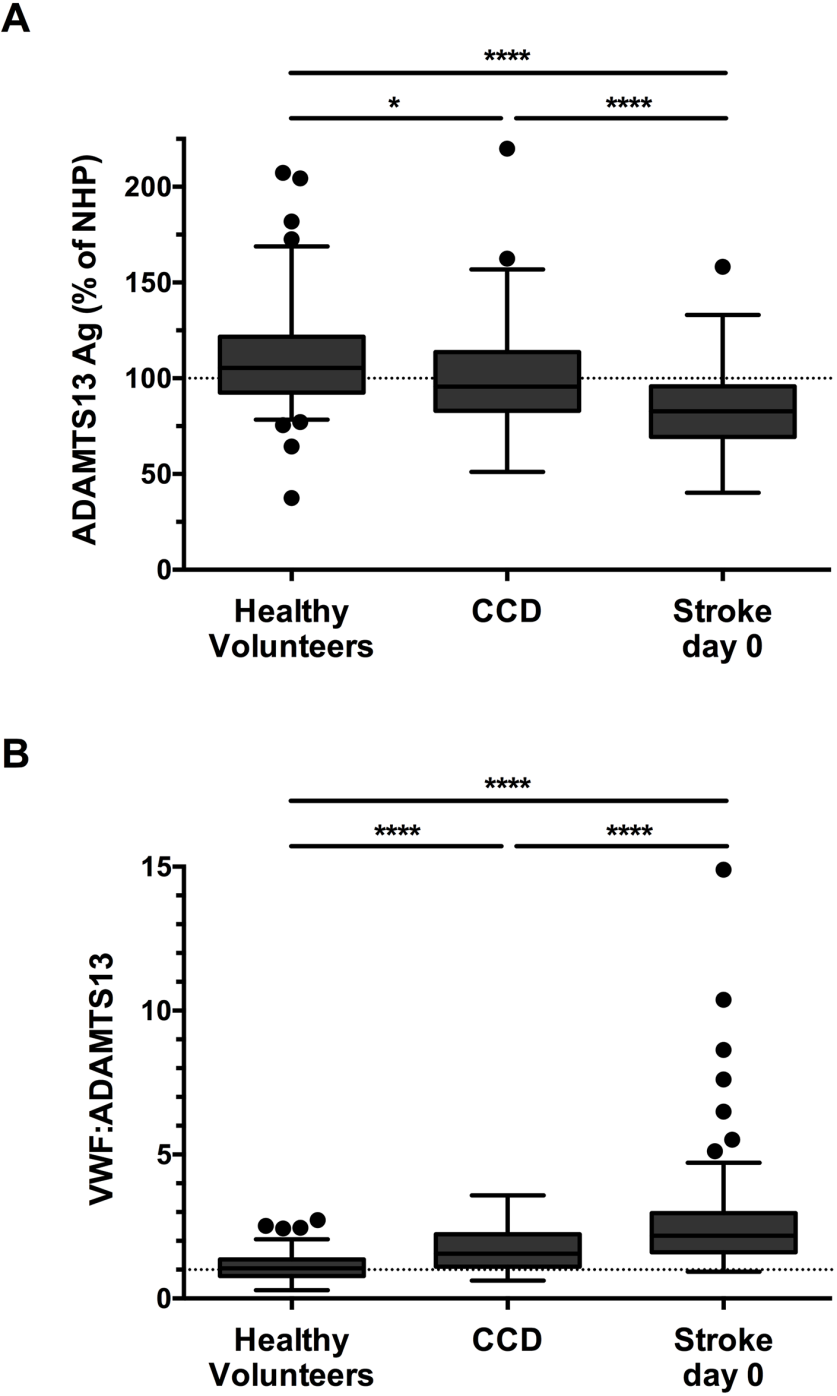
Patients with acute or chronic cerebrovascular disease have lower ADAMTS13 levels and a higher VWF:ADAMTS13 ratio compared to healthy volunteers. ADAMTS13 antigen levels from 104 patients with acute ischemic stroke, 112 patients with a chronic cerebrovascular disease (CCD) and 85 healthy volunteers, were measured. Levels of ADAMTS13 were plotted as a percentage of the level in a normal human plasma pool (NHP), consisting of pooled plasma from 20 healthy donors. VWF levels were measured previously and were used to calculate the VWF:ADAMTS13 ratios. Both ADAMTS13 levels **[A]** and VWF:ADAMTS13 ratios **[B]** are depicted in box-and-whisker plots indicating the first and third quartiles as well as the interquartile range (IQR, Tukey plot). Outliers outside the 1.5 IQR are visualized by single dots. ADAMTS13 levels and the VWF:ADAMTS13 ratios showed significant differences over the three groups. Data were analyzed using a Kruskal-Wallis test with a Dunn's multiple comparison test. *: p < 0.05; ****: p < 0.0001.

ADAMTS13 antigen levels were determined for the HV, the CCD and the acute stroke patients (Fig 1A). We found that acute stroke patients had significantly lower ADAMTS13 levels (82.6 ± 21.0%) on the day of admission compared with HV (110.6 ± 26.9%; p<0.0001). Remarkably, also CCD patients had ADAMTS13 levels significantly lower than HV (99.6 ± 24.5%; p<0.03), which were however still higher than those in acute stroke patients (p<0.0001). After adjustment for age and sex, these results remained significant (data not shown). In particular, the combination of high amounts of VWF with low levels of ADAMTS13 could form a harmful imbalance for the development of ischemic stroke. We therefore calculated the VWF:ADAMTS13 ratios in our study cohorts using previously determined VWF:Ag levels (Fig 1B).[8] Interestingly, stroke patients had a significantly higher VWF:ADAMTS13 ratio on day 0 (2.7 ± 1.9) compared with HV (1.1 ± 0.5; p<0.0001) and CCD patients (1.7 ± 0.7; p<0.0001). Also, CCD patients had significantly higher VWF:ADAMTS13 ratios compared with HV (p<0.0001). After adjustment for age and sex, these results remained significant. The time point of blood withdrawal for the stroke patients (days 0, 1 and 3) did not influence ADAMTS13 levels (p = 0.630; S1A Fig) or the VWF:ADAMTS13 ratio (p = 0.990; S1B Fig). Finally, no correlation was observed between ADAMTS13:Ag and VWF:Ag levels in acute stroke patients, CCD patients or healthy volunteers (p>0.05; S2 Fig).

### Association of ADAMTS13 and VWF:ADAMTS13 ratio with demographic and clinical parameters in acute cerebrovascular disease patients

Given the potential role of ADAMTS13 and VWF in stroke progression and severity, we correlated the levels of ADAMTS13 and the VWF:ADAMTS13 ratios in AIS and TIA patients on day 0 with key demographic and clinical parameters by univariate (Table 2) and multivariate (Table 3) analysis. In the univariate analysis, ADAMTS13 levels were only associated with sex (p = 0.018). In addition, lower ADAMTS13 levels were found in patients with the most severe stroke on admission as assessed with the National Institutes of Health Stroke Scale (NIHSS), Barthel Index (BI) and modified Ranking scale (mRS) although this did not reach statistical significance (p = 0.265; p = 0.100 and p = 0.163, respectively). Moreover, the univariate analysis revealed that VWF:ADAMTS13 ratios were significantly associated with stroke severity (NIHSS: p = 0.048; BI: p = 0.004 and mRS: p = 0.015) and stroke modality (AIS or TIA; p = 0.023) (Table 2). Also age significantly influenced VWF:ADAMTS13 ratios, with the oldest patients having the highest VWF:ADAMTS13 ratios (p = 0.038). Patients on anti-platelet treatment had higher VWF:ADAMTS13 ratios (p = 0.038) and patients who received thrombolysis had significantly lower VWF:ADAMTS13 ratios (p = 0.028).

**Table 2:** Univariate analysis of ADAMTS13 levels and the VWF:ADAMTS13 ratio with demographic and clinical parameters. Data are represented as mean ± standard deviation (SD). Data were analyzed by an independent samples Mann-Whitney U test or Kruskal-Wallis test, where applicable. (AIS: acute ischemic stroke; TIA: transient ischemic attack; NIHSS: National Institutes of Health stroke scale).

	ADAMTS13:Ag (%)	*P* value	VWF:ADAMTS13	*P* value
**Sex**				
Male	79.5 ± 20.7		2.6 ± 2.0	
Female	86.2 ± 21.0	**0.018**	2.7 ± 2.0	0.827
**Age, years**				
<55	88.0 ± 19.9		1.8 ± 0.6	
55–64	88.4 ± 24.3		2.8 ± 2.8	
65–74	83.8 ± 18.9		2.4 ± 1.0	
75–84	75.9 ± 20.1		3.1 ± 1.5	
>84	74.1 ± 19.1	0.101	3.6 ± 3.2	**0.038**
**Disease modality**				
AIS	81.7 ± 21.9		3.0 ± 2.3	
TIA	83.7 ± 20.0	0.780	2.3 ± 2.2	**0.023**
**Modified TOAST criteria**				
Cardio embolism	82.8 ± 21.6		2.6 ± 1.6	
Large-artery atherosclerosis	80.9 ± 30.7		3.4 ± 0.9	
Small-vessel occlusion	89.7 ± 24.1		2.1 ± 1.4	
Other or undetermined etiology	79.6 ± 17.4	0.668	2.9 ± 2.8	0.137
**Thrombolysis**				
Yes	84.7 ± 21.2		2.4 ± 1.3	
No	77.5 ± 19.9	0.255	3.4 ± 2.9	**0.028**
**Platelet inhibitor**				
Yes	84.5 ± 22.0		3.2 ± 2.5	
No	79.3 ± 19.0	0.198	2.4 ± 1.5	**0.038**
**NIHSS**				
0–4	84.5 ± 20.3		2.2 ± 1.2	
5–9	83.7 ± 17.1		2.7 ± 1.8	
10–15	83.5 ± 23.7		2.8 ± 0.7	
>15	66.0 ± 25.6	0.265	5.4 ± 4.9	**0.048**
**Barthel Index**				
0–30	68.8 ± 15.9		3.9 ± 2.2	
35–70	85.8 ± 18.8		2.4 ± 1.4	
>70	87.5 ± 22.9	0.100	2.1 ± 0.9	**0.004**
**Modified Ranking Scale**				
0	82.2 ± 17.1		1.9 ± 0.9	
1	82.6 ± 13.7		2.3 ± 1.1	
2	88.5 ± 29.4		2.8 ± 1.7	
3	84.4 ± 17.5		2.4 ± 0.8	
4	87.5 ± 21.4		2.5 ± 0.8	
5	63.2 ± 23.6	0.163	5.6 ± 4.5	**0.015**

**Table 3:** Multivariate analysis of ADAMTS13 levels and the VWF:ADAMTS13 ratios with demographic and clinical parameters.

	ADAMTS13			VWF:ADAMTS13		
	Coefficient	95% confidence interval	*P* value	Coefficient	95% confidence interval	*P* value
**Sex**						
Male	Reference			Reference		
Female	9.96	1.90 to 18.03	**0.016**	-0.17	-0.83 to 0.49	0.609
**Age, years**						
<55	Reference			Reference		
55–64	0.13	-13.51 to 13.77	0.985	0.57	-0.55 to 1.68	0.313
65–74	-5.25	-18.24 to 7.74	0.425	0.30	-0.77 to 1.36	0.581
75–84	-13.75	-27.50 to 0.00	0.050	0.79	-0.33 to 1.92	0.164
>84	-14.75	-31.98 to 2.48	0.092	1.01	-0.39 to 2.42	0.157
**NIHSS**						
0–4	Reference			Reference		
5–9	5.77	-6.81 to 18.35	0.365	-0.40	-1.43 to 0.63	0.443
10–15	1.04	-12.71 to 14.78	0.881	0.32	-0.80 to 1.45	0.567
>15	-13.62	-30.24 to 2.99	0.107	2.89	1.53 to 4.24	**< 0.001**
**Disease modality**	-1.77	-10.43 to 6.88	0.684	0.40	-0.31 to 1.11	0.268
**Thrombolysis**	-3.89	-14.50 to 6.72	0.469	-0.03	-0.90 to 0.83	0.942
**Platelet inhibitor**	7.47	-1.47 to 16.41	0.101	-1.04	-1.78 to -0.31	**0.006**
**CRP at admission**	-2.09	-4.52 to 0.34	0.091	0.38	0.19 to 0.58	**< 0.001**

In a multivariate analysis adjusted for age and sex we tried to identify parameters that independently influenced ADAMTS13 levels. A multivariate analysis of all variables using a linear regression model identified age (p = 0.002) and sex (p = 0.016) as independent predictors for ADAMTS13 levels (Table 3). Upon multivariate regression analysis: age (p = 0.014), CRP upon admission (p<0.001), NIHSS (p = 0.003) and the use of platelet inhibitors (p = 0.006) were identified as independent predictors of VWF:ADAMTS13 ratio’s (Table 3).

## Discussion

In this study, we report that patients with acute or chronic cerebrovascular disease have significantly lower ADAMTS13 levels. The lowest ADAMTS13 levels were found in acute ischemic stroke patients. These results further add to the notion that reduced ADAMTS13 activity is a risk factor for ischemic stroke.[10–15]

Due to the case-control design of our study we cannot draw conclusions about causality of low ADAMTS13 levels and the occurrence of ischemic stroke. Recently, however, a prospective study found that patients with the lowest ADAMTS13 activity had the highest risk for ischemic stroke, independent of other known demographic and cardiovascular risk factors.[16] Likewise, a link was recently identified between the genetic architecture of ADAMTS13, ADAMTS13 levels and stroke susceptibility.[17] These results all point towards a possible causative role for ADAMTS13 in the development of acute ischemic stroke in patients. Reduced levels of ADAMTS13 indeed could lead to a prothrombotic state allowing local accumulation of ultra large VWF at sites of vascular damage, promoting the formation of (VWF-rich) thrombo-embolic occlusions. Such local imbalance of VWF and ADAMTS13 was recently demonstrated at sites of critical coronary stenosis in patients with ST-elevation myocardial infarction.[18] The pathogenic relevance of this imbalance is further supported by our results showing that the discrepancy between acute ischemic stroke patients and healthy volunteers is even more apparent when using the ratio of VWF:ADAMTS13 as compared to ADAMTS13 alone. Similarly, in a large prospective study, Sonneveld and colleagues found that individuals who had both the lowest ADAMTS13 activity (≤25th percentile) and the highest VWF:Ag levels (≥75th percentile) had an increased risk of ischemic stroke compared with the remaining individuals.[16] More prospective studies are needed to further establish the role of low ADAMTS13 levels as a risk factor for ischemic stroke. Not surprisingly, VWF levels have been shown to independently determine ischemic stroke risk.[7] In agreement with previous studies[11,16], we did not find a correlation between VWF and ADAMTS13 levels. Hence ADAMTS13 and VWF could be independent risk factors for ischemic stroke.

Besides increasing the risk of developing ischemic stroke, reduced ADAMTS13 activity might also aggravate the progression and outcome of ischemic stroke. First, lower levels of ADAMTS13 could hamper spontaneous thrombolysis. We recently described a pro-thrombolytic effect of ADAMTS13 on VWF-rich thrombi in mice.[19] Furthermore, low ADAMTS13 levels correlated with poor response to thrombolysis in stroke patients.[20] Second, insufficient ADAMTS13 activity or high VWF activity contributes to cerebral ischemia/reperfusion injury after successful recanalization.[4–6] Our observation that the VWF:ADAMTS13 ratio was the highest in patients with the most severe stroke further underscores this importance of the VWF/ADAMTS13 axis in stroke progression and is in line with other case-control studies.[10,11]

Finally, we also report reduced levels of ADAMTS13 in patients with chronic cerebrovascular disease. CCD patients presented with extra—and/or intracranial stenosis of the larger cerebral arteries. Previously, experimental studies have identified a protective role for ADAMTS13 during the progression of atherosclerosis. Mice lacking ADAMTS13 had accelerated atherosclerotic plaque formation, which was associated with increased vascular inflammation.[21,22] Additionally, *in vivo* as well as *in vitro* experiments have demonstrated that shear gradients typically occurring at stenotic vessels, exacerbate pathological thrombus formation in a VWF-dependent manner.[23] The observed increased VWF:ADAMTS13 ratio in CCD patients further suggests the involvement of VWF and ADAMTS13 in atherosclerotic disease. However, as mentioned previously, the case-control design of our study restricts us from making conclusions about causality and future prospective clinical trials are needed to confirm these findings.

This study has some limitations. As already mentioned, because of the case–control study design of this study, it is not possible to assign causality of ADAMTS13 levels to the occurrence of stroke. Furthermore, for ethical reasons, we could not recruit patients who were unable to give informed consent. Therefore, patients with very large strokes and/or aphasia might be under-represented. Another limitation includes the relatively high proportion of TIA patients (41.9%) for which a non-vascular origin of symptoms cannot be excluded.

In conclusion, our results show the importance of ADAMTS13 in cerebrovascular disease and put forward the VWF/ADAMTS13 axis as a potential biomarker for ischemic stroke risk and severity.

## Supporting information

S1 Fig Time point of blood withdrawal did not influence ADAMTS13 levels or VWF:ADAMTS13 ratio in acute stroke patients. Levels of ADAMTS13 were plotted as a percentage of the level in a normal human plasma pool (NHP), consisting of pooled plasma from 20 healthy donors. VWF levels were measured previously and were used to calculate the VWF:ADAMTS13 ratios. Both ADAMTS13 levels **(A)** and VWF:ADAMTS13 ratios **(B)** are depicted in box-and-whisker plots indicating the first and third quartiles as well as the interquartile range (IQR, Tukey plot). Outliers outside the 1.5 IQR are visualized by single dots. Data were analyzed using a Kruskal-Wallis test with a Dunn's multiple comparison test.
(TIFF)

S2 Fig Correlation analysis between ADAMTS13 and VWF levels. A spearman correlation analysis was performed for the healthy volunteers **(A)**, the chronic cerebrovascular disease patients **(B)** and on day 0 in the acute stroke patients **(C)**. No correlation was found between plasma ADAMTS13 and VWF antigen levels.
(TIFF)

S1 Table Raw de-identified dataset. For sex: 1 indicates male, 2 indicates female; for modality: 1 indicates TIA, 2 indicates stroke.
(XLSX)

## References

[1] De Meyer SF, Stoll G, Wagner DD, Kleinschnitz C. von Willebrand factor: an emerging target in stroke therapy. Stroke. 2012;43: 599–606. 10.1161/STROKEAHA.111.628867 22180250 PMC4102321

[2] De Meyer SF, Denorme F, Langhauser F, Geuss E, Fluri F, Kleinschnitz C. Thromboinflammation in Stroke Brain Damage. Stroke. 2016;47: 1165–1172. 10.1161/STROKEAHA.115.011238 26786115

[3] Chauhan AK, Motto DG, Lamb CB, Bergmeier W, Dockal M, Plaimauer B, et al Systemic antithrombotic effects of ADAMTS13. Journal of Experimental Medicine. 2006;203: 767–776. 10.1084/jem.20051732 16533881 PMC2118248

[4] Kleinschnitz C, De Meyer SF, Schwarz T, Austinat M, Vanhoorelbeke K, Nieswandt B, et al Deficiency of von Willebrand factor protects mice from ischemic stroke. Blood. 2009;113: 3600–3603. 10.1182/blood-2008-09-180695 19182208

[5] Zhao BQ, Chauhan AK, Canault M, Patten IS, Yang JJ, Dockal M, et al von Willebrand factor-cleaving protease ADAMTS13 reduces ischemic brain injury in experimental stroke. Blood. 2009;114: 3329–3334. 10.1182/blood-2009-03-213264 19687510 PMC2759655

[6] De Meyer SF, Schwarz T, Deckmyn H, Denis CV, Nieswandt B, Stoll G, et al Binding of von Willebrand factor to collagen and glycoprotein Ibalpha, but not to glycoprotein IIb/IIIa, contributes to ischemic stroke in mice—brief report. Arteriosclerosis, Thrombosis, and Vascular Biology. 2010;30: 1949–1951. 10.1161/ATVBAHA.110.208918 20616311

[7] Sonneveld MAH, de Maat MPM, Leebeek FWG. Von Willebrand factor and ADAMTS13 in arterial thrombosis: a systematic review and meta-analysis. Blood Rev. 2014;28: 167–178. 10.1016/j.blre.2014.04.003 24825749

[8] Kraft P, Drechsler C, Gunreben I, Nieswandt B, Stoll G, Heuschmann PU, et al Von Willebrand factor regulation in patients with acute and chronic cerebrovascular disease: a pilot, case-control study. PLoS ONE. 2014;9: e99851 10.1371/journal.pone.0099851 24937073 PMC4061052

[9] Feys HB, Liu F, Dong N, Pareyn I, Vauterin S, Vandeputte N, et al ADAMTS-13 plasma level determination uncovers antigen absence in acquired thrombotic thrombocytopenic purpura and ethnic differences. J Thromb Haemost. 2006;4: 955–962. 10.1111/j.1538-7836.2006.01833.x 16689741

[10] Bongers TN, de Bruijne ELE, Dippel DWJ, de Jong AJ, Deckers JW, Poldermans D, et al Lower levels of ADAMTS13 are associated with cardiovascular disease in young patients. Atherosclerosis. 2009;207: 250–254. 10.1016/j.atherosclerosis.2009.04.013 19439298

[11] Andersson HM, Siegerink B, Luken BM, Crawley JTB, Algra A, Lane DA, et al High VWF, low ADAMTS13, and oral contraceptives increase the risk of ischemic stroke and myocardial infarction in young women. Blood. 2012;119: 1555–1560. 10.1182/blood-2011-09-380618 22110247

[12] Lambers M, Goldenberg NA, Kenet G, Kirkham FJ, Manner D, Bernard T, et al Role of reduced ADAMTS13 in arterial ischemic stroke: A Pediatric Cohort Study. Ann Neurol. 2012;73: 58–64. 10.1002/ana.23735 23225307 PMC3703929

[13] Allie S, Stanley A, Bryer A, Meiring M, Combrinck MI. High levels of von Willebrand factor and low levels of its cleaving protease, ADAMTS13, are associated with stroke in young HIV-infected patients. Int J Stroke. 2015;10: 1294–1296. 10.1111/ijs.12550 26121272

[14] McCabe DJH, Murphy SJX, Starke R, Harrison P, Brown MM, Sidhu PS, et al Relationship between ADAMTS13 activity, von Willebrand factor antigen levels and platelet function in the early and late phases after TIA or ischaemic stroke. Journal of the Neurological Sciences. 2015;348: 35–40. 10.1016/j.jns.2014.10.035 25498844

[15] Qu Le, Jiang M, Qiu W, Lu S, Zhao Y, Xia L, et al Assessment of the Diagnostic Value of Plasma Levels, Activities, and Their Ratios of von Willebrand Factor and ADAMTS13 in Patients with Cerebral Infarction. Clin Appl Thromb Hemost. 2016;22: 252–259. 10.1177/1076029615583347 25916953

[16] Sonneveld MAH, de Maat MPM, Portegies MLP, Kavousi M, Hofman A, Turecek PL, et al Low ADAMTS13 activity is associated with an increased risk of ischemic stroke. Blood. 2015;126: 2739–2746. 10.1182/blood-2015-05-643338 26511134

[17] Stoll M, Rühle F, Witten A, Barysenka A, Arning A, Strauss C, et al Rare Variants in the ADAMTS13 Von Willebrand Factor-Binding Domain Contribute to Pediatric Stroke. Circ Cardiovasc Genet. 2016;9: 357–367. 10.1161/CIRCGENETICS.115.001184 27412500

[18] Pedrazzini G, Biasco L, Sulzer I, Anesini A, Moccetti T, Kremer Hovinga JA, et al Acquired intracoronary ADAMTS13 deficiency and VWF retention at sites of critical coronary stenosis in patients with STEMI. Blood. 2016;127: 2934–2936. 10.1182/blood-2015-12-688010 27034431

[19] Denorme F, Langhauser F, Desender L, Vandenbulcke A, Rottensteiner H, Plaimauer B, et al ADAMTS13-mediated thrombolysis of t-PA-resistant occlusions in ischemic stroke in mice. Blood. 2016;127: 2337–2345. 10.1182/blood-2015-08-662650 26929275

[20] Bustamante A, Llombart V, Boada C, Penalba A, Simats A, García-Berrocoso T, et al Abstract 189: ADAMTS13 Activity Predicts Response to Thrombolysis in the Acute Stroke Setting. Stroke. 2015;46: A189.

[21] Gandhi C, Khan MM, Lentz SR, Chauhan AK. ADAMTS13 reduces vascular inflammation and the development of early atherosclerosis in mice. Blood. 2012;119: 2385–2391. 10.1182/blood-2011-09-376202 22123843 PMC3311260

[22] Jin S-Y, Tohyama J, Bauer RC, Cao NN, Rader DJ, Zheng XL. Genetic ablation of Adamts13 gene dramatically accelerates the formation of early atherosclerosis in a murine model. Arteriosclerosis, Thrombosis, and Vascular Biology. 2012;32: 1817–1823. 10.1161/ATVBAHA.112.247262 22652598 PMC3422623

[23] Westein E, van der Meer AD, Kuijpers MJE, Frimat J-P, van den Berg A, Heemskerk JWM. Atherosclerotic geometries exacerbate pathological thrombus formation poststenosis in a von Willebrand factor-dependent manner. Proceedings of the National Academy of Sciences. 2013;110: 1357–1362. 10.1073/pnas.1209905110 23288905 PMC3557050

