# Supplementary figures and images for: Reduced ADAMTS13 levels in patients with acute and chronic cerebrovascular disease
Source: PLoS One. 2017 Jun 7;12(6):e0179258. doi: 10.1371/journal.pone.0179258 (PMC5462472; doi:10.1371/journal.pone.0179258)

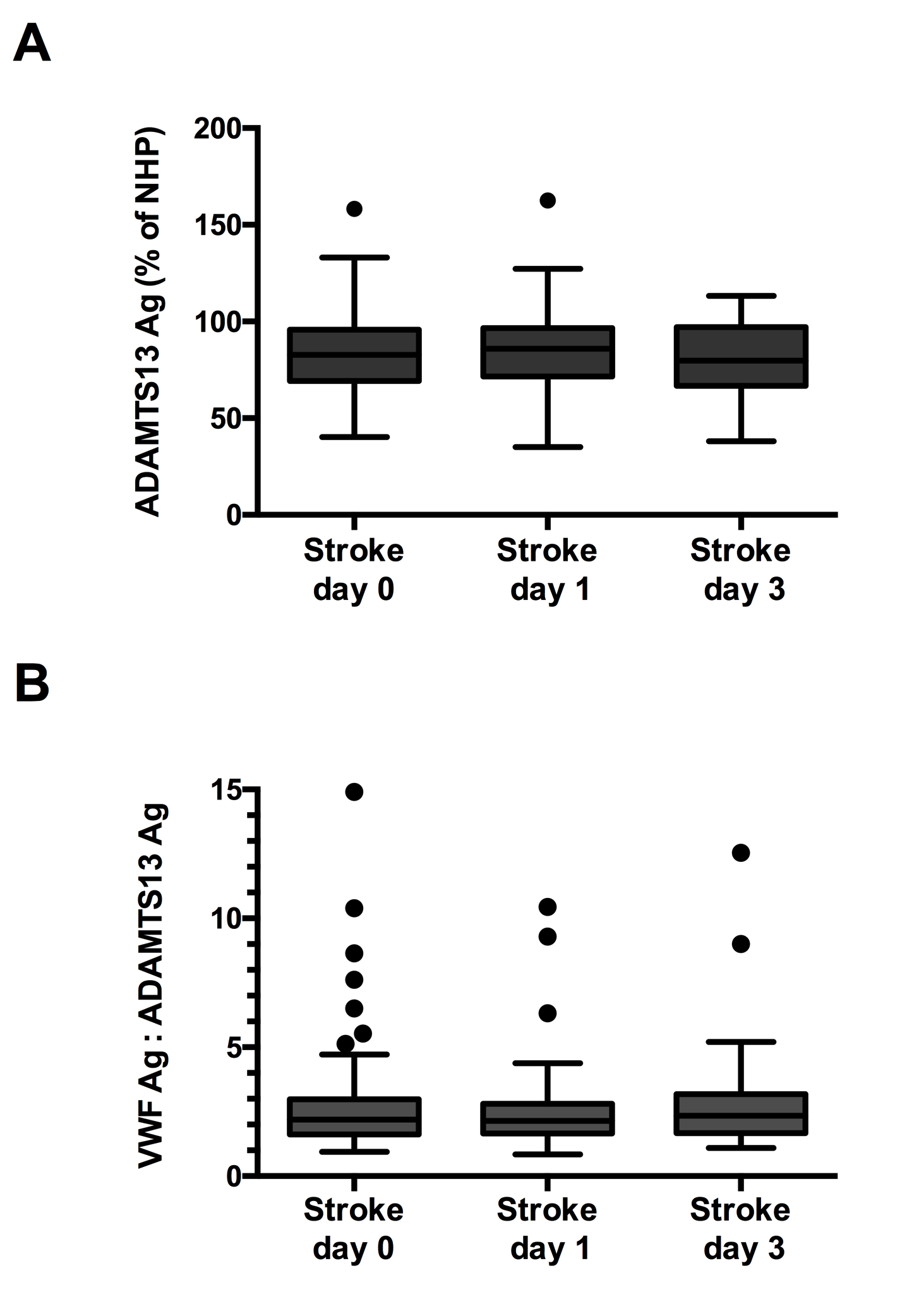

Supplement: S1 Fig — Levels of ADAMTS13 were plotted as a percentage of the level in a normal human plasma pool (NHP), consisting of pooled plasma from 20 healthy donors. VWF levels were measured previously and were used to calculate the VWF:ADAMTS13 ratios. Both ADAMTS13 levels (A) and VWF:ADAMTS13 ratios (B) are depicted in box-and-whisker plots indicating the first and third quartiles as well as the interquartile range (IQR, Tukey plot). Outliers outside the 1.5 IQR are visualized by single dots. Data were analyzed using a Kruskal-Wallis test with a Dunn's multiple comparison test. (TIFF) [file pone.0179258.s001.tiff]

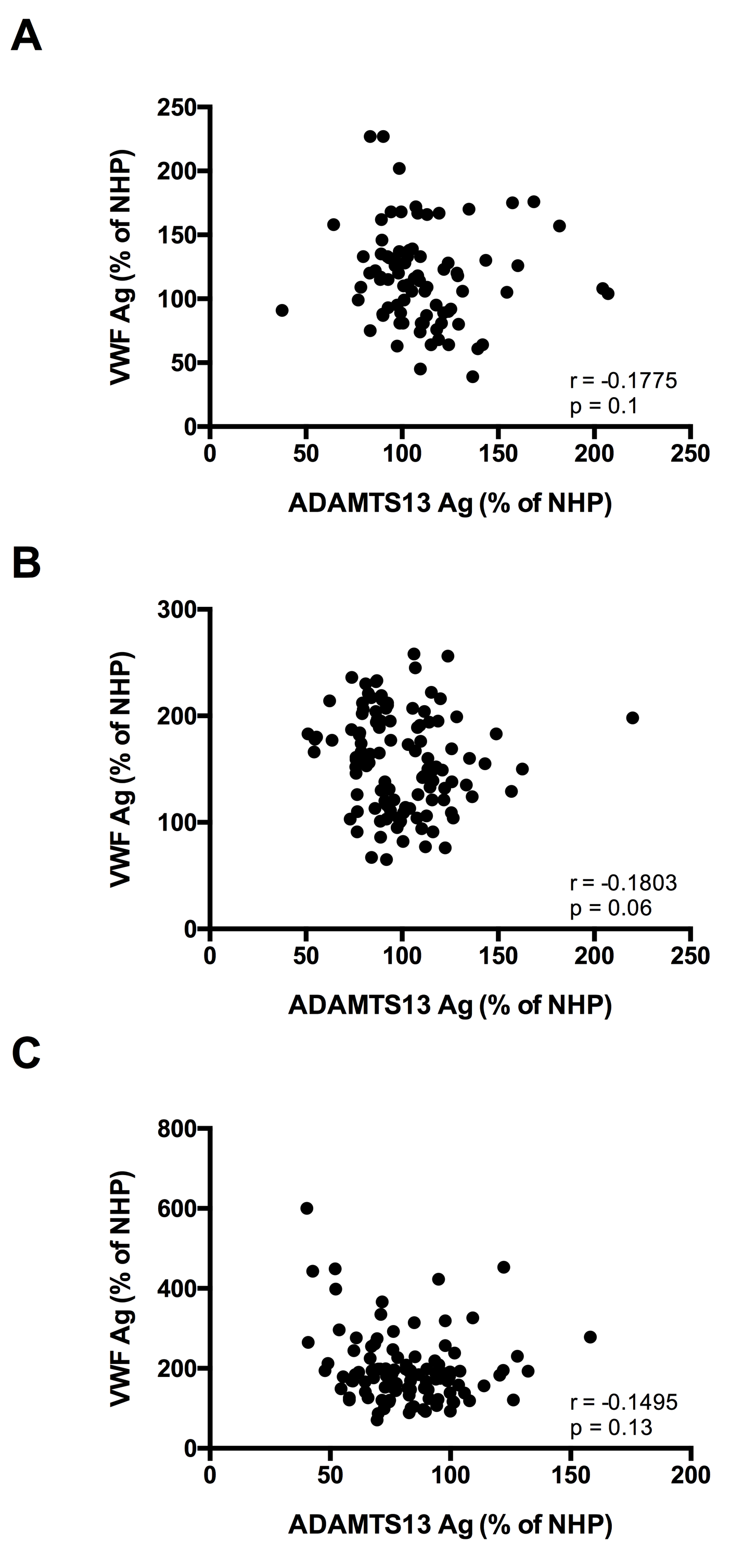

Supplement: S2 Fig — A spearman correlation analysis was performed for the healthy volunteers (A), the chronic cerebrovascular disease patients (B) and on day 0 in the acute stroke patients (C). No correlation was found between plasma ADAMTS13 and VWF antigen levels. (TIFF) [file pone.0179258.s002.tiff]
